# Synthesis and Effect of Encapsulating Rejuvenator Fiber on the Performance of Asphalt Mixture

**DOI:** 10.3390/ma12081266

**Published:** 2019-04-17

**Authors:** Benan Shu, Shiwen Bao, Shaopeng Wu, Lijie Dong, Chao Li, Xu Yang, José Norambuena-Contreras, Quantao Liu, Qing Wang

**Affiliations:** 1 State Key Laboratory of Silicate Materials for Architectures, Wuhan University of Technology, Wuhan 430070, China; shuba@whut.edu.cn (B.S.); baosw@whut.edu.cn (S.B.); lic@whut.edu.cn (C.L.); 2 State Key Laboratory of Advanced Technology for Materials Synthesis and Processing, and School of Materials Science and Engineering, Wuhan University of Technology, Wuhan 430070, China; lijie@whut.edu.cn (L.D.); 254546@whut.edu.cn (Q.W.); 3 Department of Civil Engineering, Monash University, Clayton, VIC 3800, Australia; xu.yang@monash.edu; 4 LabMAT, Department of Civil and Environmental Engineering, University of Bío-Bío, Concepción 4030000, Chile; jnorambuena@ubiobio.cl

**Keywords:** asphalt mixture, encapsulated rejuvenator, road performance, self-healing

## Abstract

The idea of prolonging the service life of asphalt mixture by improving the self-healing ability of asphalt has received extensive attention in recent years. In view of this, this work synthesized three kinds of encapsulating rejuvenator fibers to improve self-healing properties of asphalt mixtures. A series of characterizations were performed to study the morphology, chemical structure and thermal stability of the three kinds of fibers. Subsequently, the road performance of asphalt mixture containing the fiber were investigated, which included high and low temperature, water sensitivity and fatigue performances. Finally, the self-healing performance of asphalt mixture containing the fiber was investigated by 3PB test. The results revealed that the three kinds of encapsulating rejuvenator fibers were successfully synthesized. The fibers had excellent thermal stability, which met temperature requirements in the mixing and compaction process of asphalt mixtures. Road performance of asphalt mixture containing the fiber met the requirements. Self-healing ability of asphalt mixture containing the fiber was improved. Synergistic action of temperature and rejuvenator could further significantly improve the self-healing ability of the asphalt mixture.

## 1. Introduction

Asphalt mixture, which is one of the most widely used pavement materials worldwide, contains bitumen, filler, fine aggregate, and coarse aggregate. After several years of service, cracking, as one of the most common diseases, will be generated in the interior of asphalt pavements due to the hardening and brittleness of aged asphalt, vehicle loading and so on [1,2,3,4]. Although asphalt binder has inherent self-healing ability, the ability was limited under the action of continuous traffic loading, moisture ingress and other factors. Thus, continuous development of cracks will lead to bitumen pavement failure [5,6,7,8,9]. It is urgent to improve the self-healing of asphalt to extend the service life and reduce maintenance cost of asphalt pavement. 

At present, there are several ways to improve the self-healing capability of asphalt, which include nanoparticles [10,11], electromagnetic induction [12,13] and microwave heating [14,15]. The application of these technologies could effectively improve the self-healing of asphalt. In recent years, capsule method have attracted wide attention because of its crack response ability. Cracks can induce capsules ruptured, which we called it “crack response ability”. Under the action of capillary, the healing agent that was encapsulated in capsules thus fills up cracks and heal the cracks [16]. 

The mechanism by which capsules improve the self-healing ability of asphalt is that: when the cracks inside an asphalt mixture develop to the surface of capsules, capsules were ruptured under the action of stress concentration. Because of capillary siphon, the encapsulated rejuvenator flows out and fills up the micro-cracks. Rejuvenator can supplement the light components that was lost in the service of asphalt binder [17,18]. In addition, the infiltration and diffusion of rejuvenator can significantly reduce the viscosity of asphalt binder around the cracks, and then the flow ability of asphalt is improved [19]. Therefore, the addition of encapsulating rejuvenator capsules could improve the self-healing properties of asphalt mixture. 

A lot of studies have been done to synthesize various types of encapsulations containing rejuvenator to improve self-healing capability of asphalt [20,21,22]. For example, Sun et al. [23,24] synthesized melamine-urea-formaldehyde microcapsules by an in situ polymerization method. The optimal capsules were obtained by adjusting the parameters, which include the ratio of core to shell materials, reaction temperature, type and content of emulsifier. The optimal capsule met the temperature requirement in mixing and compaction process of asphalt mixture. In addition, the four-point bending fatigue test of asphalt mixture revealed that the fatigue life of asphalt mixture containing 3% microcapsules was double than that of asphalt mixture without microcapsules. Li et al. [25] successfully synthesized urea–formaldehyde microcapsules encapsulating asphalt rejuvenator. The parameters that affect structure and size of microcapsules were considered, for example, the stirring speed and reaction time. In addition, the healing efficiency of asphalt with the content of 0.3% microcapsules increased by 38.67%. Zhang et al. [26] synthesized urea formaldehyde self-healing microcapsules, and then the rheological properties of asphalt binder with the microcapsules was deeply studied. 

Norambuena-Contreras [27] fabricated multinuclear calcium alginate capsules encapsulating sunflower oil by ionotropic gelation. The oil content in capsules could be easily controlled. Furthermore, the factors that can affect the self-healing efficiency of asphalt mixture were investigated, for example the aging effect, addition order of capsules and temperature. The result showed that the self-healing ability of asphalt mixtures with 0.5% capsules is several times higher than that of asphalt mixtures without capsules. The author’s group synthesized calcium alginate microcapsules and compartmented calcium alginate/silica fiber encapsulating rejuvenator by using a microfluidic device [28,29]. The encapsulations had excellent thermal and mechanical properties. Furthermore, experimental results confirmed from the view of the macroscopic and microscopic scales that the addition of encapsulations could improve the self-healing ability of asphalt. 

Up to now, most studies have focused on the synthesis of encapsulations and the self-healing performance of asphalt mixtures containing encapsulations, and few studies concerned the road performance of asphalt mixtures containing encapsulations. Before studying the self-healing performances, the road performance of asphalt mixture with encapsulations should meet the technology requirements. For example, the high and low temperature performances, water sensitivity and fatigue ability. In view of this, the three kinds of fibers encapsulating asphalt rejuvenator were synthesized in this study. Road performances of asphalt mixture with different kinds of fiber were studied. Subsequently, three-point bending test was performed to evaluate the self-healing capability of asphalt mixture.

## 2. Materials and Test Methods

### 2.1. Gradation Design of Asphalt Mixture

AC-13 asphalt mixture was designed in this work. Aggregate gradation is shown in Table 1. 70# bitumen was used in this work, and its penetration (20 °C, 0.1 mm) and softening point were 68.7 and 48.5 °C respectively. Its ductility (15 °C) was larger than 100 cm. Basalt was used as aggregate. The optimum bitumen content and the content of the fiber were 4.7% and 0.235%, respectively, based on Marshall design method. air void (VV), voids in mineral aggregate (VMA) and voids filled with asphalt (VFA) were 3.9%, 14.5% and 73.1%, respectively.

### 2.2. Synthesis of the Three Kinds of Fibers

Alginate, dehydrated calcium chloride, silica (SiO_2_) nanoparticles, graphene oxide (GO) and asphalt rejuvenator were purchased from SINOPHARM GROUP CO. LTD. Asphalt rejuvenator was mainly composed of 67.4% aromatics and 21.07% saturates. 

Alginate solution (2 weight% alginate) was prepared to synthesize calcium alginate fiber encapsulating asphalt rejuvenator. Alginate/SiO_2_ (alginate: SiO_2_ = 1:1) solution was prepared to synthesize calcium alginate/SiO_2_ composite fiber encapsulating rejuvenator. Alginate/GO (alginate: GO = 1:10) solution was prepared to synthesize calcium alginate/GO composite fiber encapsulating rejuvenator. The purpose of synthesizing the three kinds of fibers is that: the synthesis of calcium alginate/SiO_2_ composite fiber is going to improve thermal and mechanical properties, and decrease the leakage of calcium alginate fiber; the synthesis of calcium alginate/GO composite fiber is going to combine the advantages of the induction heating method and the capsule method. The fiber can be heated by microwave to increase the temperature of asphalt, so as to achieve the purpose of quickly healing cracks inside asphalt. Further, the asphalt can be rejuvenated by the encapsulated rejuvenator. Therefore, calcium alginate/GO composite fiber can make the cracks inside the asphalt double healed.

The three kinds of fibers were synthesized by using a self-assembled microfluidic device. The synthetic process was shown in a previous study [23]. 

### 2.3. Characterization of the Three Kinds of Fibers

Morphology of the three kinds of fibers was characterized by scanning electron microscope (SEM) test. SEM test was conducted on an S4800 machine. Before SEM test, the specimens were sprayed with gold for 50 s. 

Chemical structure of the three kinds of fibers was evaluated by Raman spectra test. An InVia instrument with a wavelength ranging from 200 to 2000 cm^−1^ was used to complete Raman spectra test.

Thermal properties of the three kinds of fibers were tested by thermogravimetric analyzer (TGA) experiment. The testing temperature raised from 30 to 600 °C with a heating rate of 10 °C/min. 

### 2.4. Road Performances of Asphalt Mixture with the Fibers

Rutting test, water stability test, freeze-thaw splitting test and low temperature bending test of asphalt mixture were carried out according to the specification “Standard Test Methods of Bitumen and Bituminous Mixtures for Highway Engineering JTG E20-2011”. 

Rutting test was conducted on a YLDCZ-6S machine with the testing temperature of 60 °C. The wheel speed was 21 times/min, and loading was 0.7 MPa. The length, width and height of specimen were 300 mm, 300 mm and 50 mm, respectively. Before the testing, the sample was kept 5 h at 60 °C. The dynamic stability (DS) was defined as follows:

(1) $$\mathrm{DS}=15\times N/\left(d_{60}-d_{45}\right)$$

where N=42 cycles/min, which means the wheel tracking speed; $d_{60}$ and $d_{45}$ are the rutting depth at the wheel tracking time of 45 min and 60 min, respectively.

The low temperature crack resistance of asphalt mixture was evaluated by flexural strength, flexural strain and flexural modulus. The length, width and height of specimen were 250 mm, 30 mm and 35 mm, respectively. The test temperature was −10 °C and the loading rate was 50 mm/min.

The water stability of asphalt mixture with the fibers was evaluated by freeze-thaw split strength ratio (TSR). For that, the specimen was firstly freezing at −18 °C for 16h, and then suffered a water bath for 24 h at 60 °C.

(2) $$\mathrm{TSR}=\frac{TS_{2}}{TS_{1}}\times 100\%$$

where $TS_{2}$ was splitting tensile strength of the specimen after freeze-thaw experiment; $TS_{1}$ was splitting tensile strength of the specimen before freeze-thaw experiment.

Four-point bending fatigue test was carried out to study the fatigue performance of asphalt mixture containing the fiber. Beams with length, width and height of 400 mm, 50 mm and 50 mm respectively were used. The test temperature and working frequency were 20 °C and 30 Hz respectively. 

### 2.5. Self-Healing Capability of Asphalt Mixture with the Fibers

Three-point bending test on the asphalt mixture beams with length, width and height of 95 mm, 40 mm and 50 mm respectively, was performed by using a universal testing machine (UTM-25). Before the test, the specimens were kept at −10 °C for 4 hours. Then the specimens were tested at −10 °C with a loading rate of 0.5 mm/min. After that, the two parts of a tested specimen were rejoined by three rubber bands. Healing temperature and healing time were 30 °C and 3 days respectively. A microwave oven with the power of 800 W was used to complete microwave heating test. The self-healing ability of each asphalt mixture beam was quantified by the Healing Index (HI) according to Equation (3).

(3) $$HI=\left(\frac{F_{r}}{F_{i}}\right)\times 100$$

where, $F_{r}$ is recovery of peak strength of an asphalt beam after healing rest, kN; $F_{i}$ is the initial peak strength of the same asphalt beam, kN.

Types symbology of fibers and asphalt mixtures is shown in Table 2. A1 was the asphalt mixture without fiber. Three sets of replicate samples were tested in each experiment.

## 3. Results and Discussion

### 3.1. Characterization of Three Kinds of Self-Healing Fibers

#### 3.1.1. Morphology 

**Figure 1:**
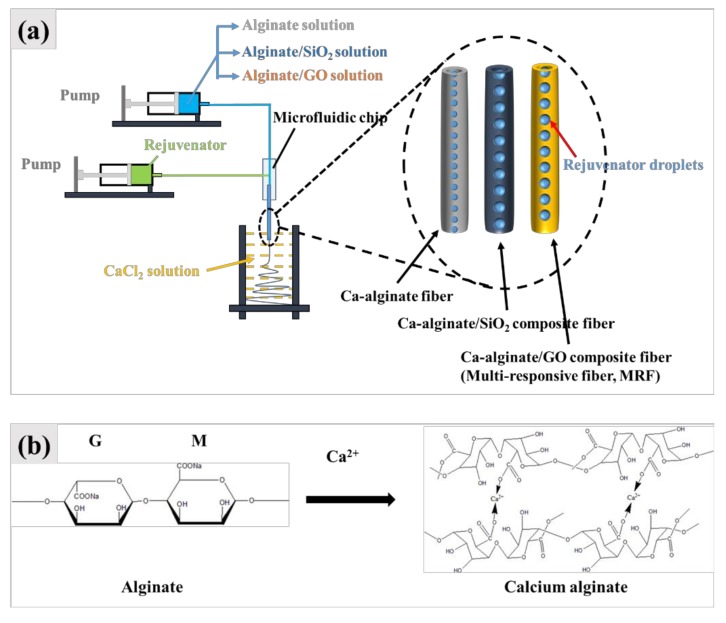
Synthesis process (**a**) and synthesis mechanism (**b**) of the three kinds of fibers.

Figure 1 shows the synthesis process and synthesis mechanism of the three kinds of self-healing fibers. Alginate is composed of a series of G units and M units. Sodium ions on G units can be rapidly replaced by calcium ions to form calcium alginate gels. The whole reaction process is completed in an instant, so, nano SiO_2_ particles and graphene oxide particles can be completely incorporated into calcium alginate gel. The three kind of self-healing fibers, which include Ca–alginate fiber, Ca–alginate/SiO_2_ composite fiber and Ca–alginate/GO composite fiber were thus fabricated.

**Figure 2:**
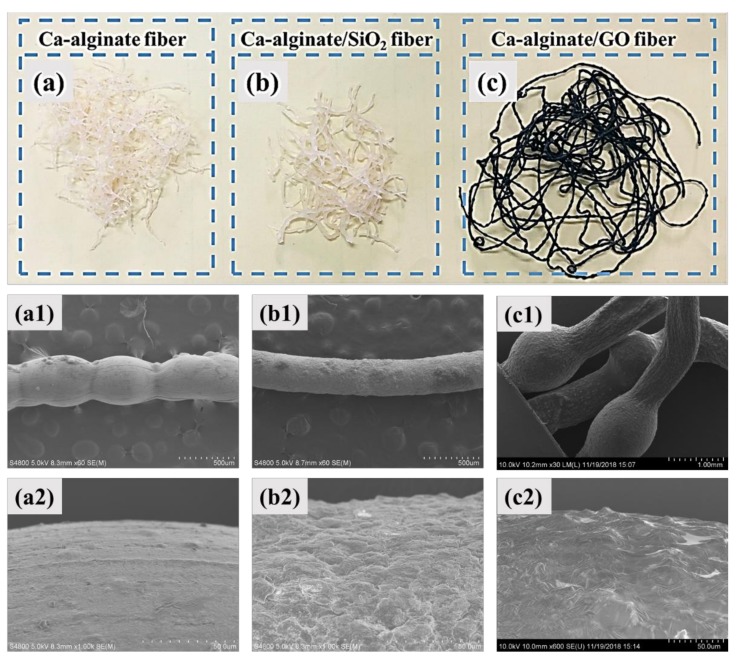
Morphology of the three kinds of self-healing fiber: Optical morphologyof Ca-alginate fiber (**a**), Ca-alginate/SiO_2_ fiber (**b**) and Ca-alginate/GO fiber (**c**); Micromorphology of Ca-alginate fiber (**a1**,**a2**), Ca-alginate/SiO_2_ fiber (**b1**,**b2**) and Ca-alginate/GO fiber (**c1**,**c2**).

The morphology of three kinds of self-healing fibers is shown in Figure 2. It can be seen from Figure 2a–c that AF and ASF had a light-yellow optical appearance, while MRF had a black appearance. From Figure 2(a1–c1), it can be observed that the wall of three kinds of fibers was intact and there were no visible holes and cracks, which indicated that rejuvenator could be completely encapsulated inside the fibers, and without leakage. The diameter of the fibers was about 500 μm. Figure 2(a2–c2) reveal that the surface of AF was the smoothest. ASF fibers had an uneven surface with many small bulges, which was attributed to the incorporation of partially agglomerated SiO_2_ particles. A large number of regular folds appeared on the surface of MRF, which were peculiar to the specific structure of graphene. Over all, the three kinds of self-healing fibers encapsulating rejuvenator were successfully synthesized from the analysis of morphology.

#### 3.1.2. Chemical Structure 

**Figure 3:**
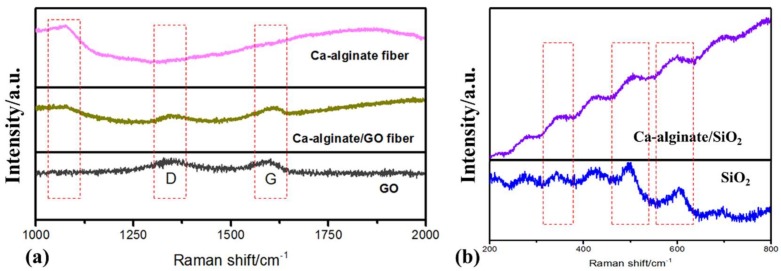
Raman test of the three kinds of fibers: (**a**) Ca–alginate fiber and Ca–alginate/GO fiber; (**b**) Ca–alginate/SiO_2_ fiber.

Raman test was conducted to confirm the successful synthesis of the three kinds of self-healing fibers. Figure 3a revealed that GO had two characteristic peaks, namely peak D and peak G. The two peaks didn’t appear in the Raman spectra of calcium alginate fiber, while appeared in Ca–alginate/GO fiber Raman spectra. A characteristic peak at 1070 cm^−1^ appeared in both Ca–alginate fiber and Ca–alginate/GO fiber. The three kinds of characteristic peaks appeared in the Raman spectra of Ca–alginate/GO fiber, which indicated that Ca–alginate/GO fiber was successfully synthesized. Similarly, SiO_2_ had a series of characteristic peaks in the Raman shift from 200 cm^−1^ to 800 cm^−1^ in Figure 3b. These peaks also appeared at the same wavelengths in Raman spectrum of Ca–alginate/SiO_2_ fiber, which revealed that SiO_2_ was successfully incorporated inside the Ca–alginate structure, and Ca–alginate/SiO_2_ composite fiber was successfully synthesized.

#### 3.1.3. Thermal Stability 

**Figure 4:**
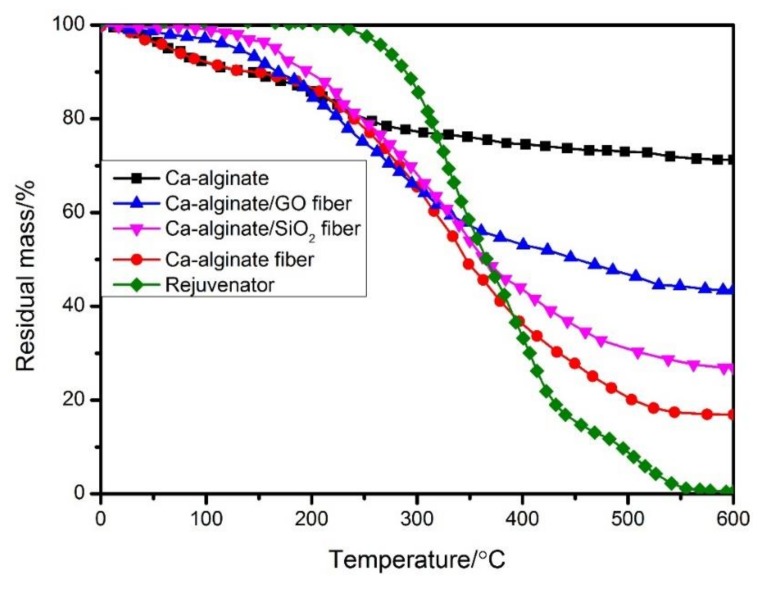
Thermal stability of the three kinds of fiber.

The thermal stability of three kinds of self-healing fibers was studied by TGA test and the results is shown in Figure 4. It can be seen that the initial decomposition temperature of rejuvenator was about 250 °C, which means that the rejuvenator meet the temperature requirement in the mixing and compaction process of asphalt mixture. Between 100 °C and 180 °C, the loss mass of Ca–alginate self-healing fiber was attributed to degradation of crystalline water and other components of calcium alginate wall. The decomposition mass and decomposition rate of ASF and MRF were both significantly lower than those of AF, which indicated that the addition of nano-SiO_2_ and GO particles could improve the thermal stability of Ca–alginate fiber. The loss mass of fiber between 200 and 400 °C was mainly attributed to the decomposition of rejuvenator and calcium alginate materials. Based on the residual mass at 600 °C, the relative mass of encapsulated rejuvenator could be calculated out. The relative mass of rejuvenator that was encapsulated in AF, ASF and MRF were 56.65%, 47.25% and 32.94% respectively. Based on the above analysis, the three kinds of self-healing fiber meet the temperature requirement in the mixing and compaction process of asphalt mixture. 

### 3.2. Road Performance of the Asphalt Mixture with Fibers

#### 3.2.1. High-Temperature Performance

**Figure 5:**
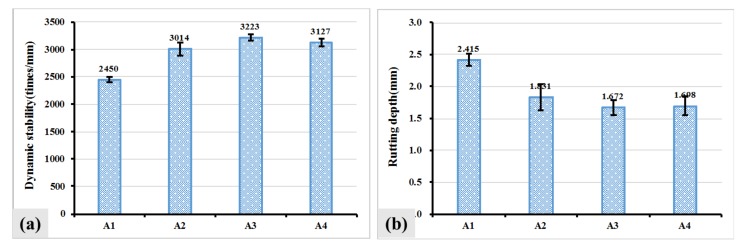
DS and rutting depth of asphalt mixture with the fiber: DS (**a**) and RD (**b**) of asphalt mixture.

The high temperature performance of asphalt mixture containing the different kinds of fiber was investigated by wheel tracking test and the result is shown in Figure 5. Dynamic stability (DS) and rutting depth (RD) were tested to evaluate high temperature anti-rutting ability of asphalt mixture. The larger DS and smaller rutting depth, the better anti-rutting ability asphalt mixture has. It can be seen that the addition of fibers led to an improved DS and decreased rutting depth. For example, the DS of A2, A3 and A4 increased from 2450 times/mm to 3014 times/mm, 3223 times/mm and 3127 times/mm respectively. The rutting depth of A2, A3 and A4 decreased from 2.415 mm to 1.831 mm, 1.672 mm and 1.698 mm respectively. The improved anti-rutting ability may be attributed to that the entanglement of fibers in asphalt mixture reduced the content of free asphalt. 

#### 3.2.2. Low Temperature Performance

**Figure 6:**
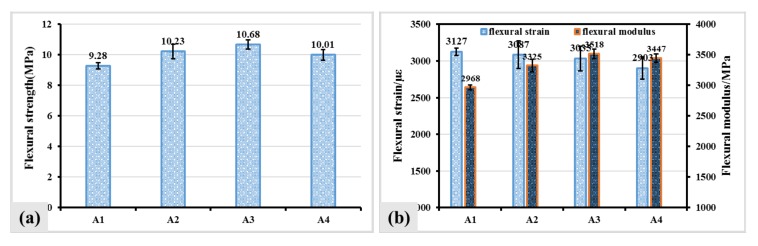
Flexural strength (**a**), Flexural strain and flexural modulus (**b**) of asphalt mixture containing the fiber.

Low temperature bending test was conducted, and flexural strength, flexural strain and flexural modulus were obtained to evaluate low temperature anti-crack ability of asphalt mixture containing the fiber. Figure 6a revealed that the addition of the fiber increased flexural strength of asphalt mixture. The flexural strength of A2, A3 and A4 increased from 9.28 MPa to 10.23 MPa, 10.68 MPa and 10.01 MPa, respectively. It can be seen from Figure 6b that the flexural strain of asphalt mixture containing the fiber was slightly smaller than that of asphalt mixture without fibers. Nevertheless, the flexural strains of all asphalt mixtures containing the fiber were larger than 2800με, which indicates that the flexural strain of asphalt mixture containing the fiber meets the requirements. In addition, the addition of the fiber could improve the flexural modulus of asphalt mixture. The flexural modulus of A2, A3 and A4 increased from 2968 MPa to 3325 MPa, 3518 MPa and 3447MPa, respectively.

#### 3.2.3. Water Sensitivity

**Figure 7:**
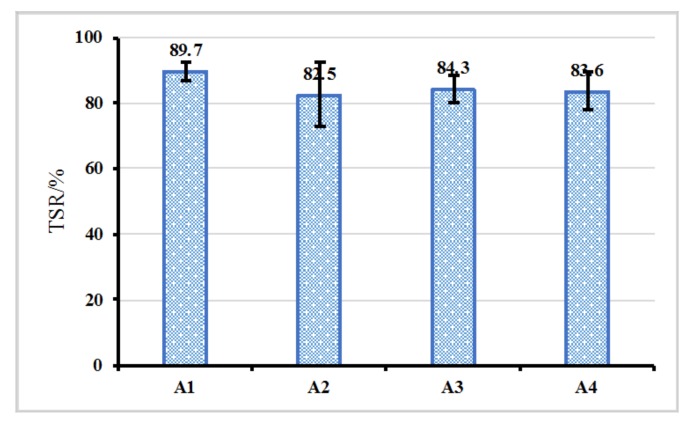
TSR of asphalt mixture with the fiber.

Freeze-thaw splitting test was performed to evaluate the water sensitivity of asphalt mixture containing the fibers, and the result is shown in Figure 7. It can be observed that the TSR of asphalt mixture slightly decreased when the fiber was added. In details, the TSR of A2, A3 and A4 decreased from 89.7% to 82.5%, 84.3% and 83.6%, respectively. The slightly decreased TSR may be attributed to the relatively strong hydrophilicity of the fiber. Nevertheless, the TSR of asphalt mixture with the fiber was still larger than 75%. Thus, moisture stability of asphalt mixture with the fiber met the requirement (according to the specification “Technical Specification for Construction of Highway Asphalt Pavements JTG F40-2004”).

#### 3.2.4. Fatigue Performance

**Figure 8:**
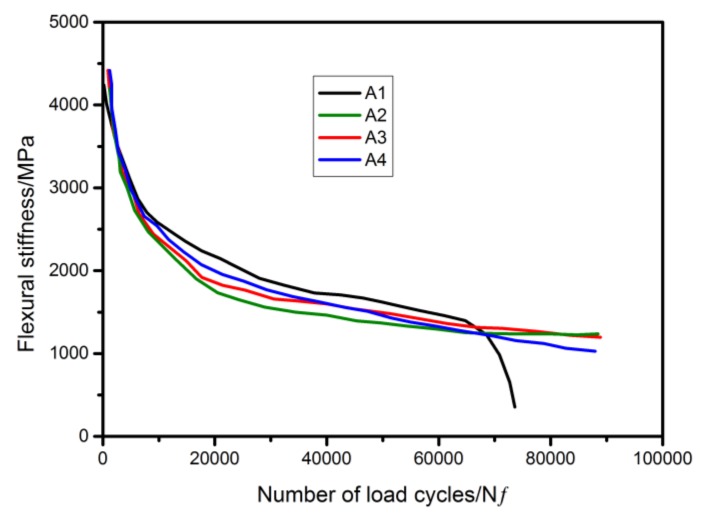
Fatigue performance of asphalt mixture with the fibers.

Four-point bending fatigue test was conducted to evaluate fatigue performance of asphalt mixture with the fiber. It can be seen from Figure 8 that there was no obvious difference in initial stiffness for asphalt mixture with, and without the fiber, which meant that the fibers could remain intact in asphalt mixture after the mixing and compaction process. The stiffness of asphalt mixture with the fiber was smaller than that of asphalt mixture without the fiber from 10,000 numbers of load cycles to 60,000 numbers of load cycles, which indicated that the fiber was ruptured, rejuvenator that was encapsulated in the fiber flowered out, and then rejuvenated and softened bitumen. Fatigue failure of asphalt mixture without the fiber appeared when the number of load cycles was larger than 60,000. While due to the regeneration of rejuvenator, asphalt mixture with the fiber could continue to withstand fatigue loading. Thus the addition of the fibers could prolong the fatigue life of asphalt mixture.

### 3.3. Self-Healing Capacity of the Asphalt Mixture with Fibers

**Figure 9:**
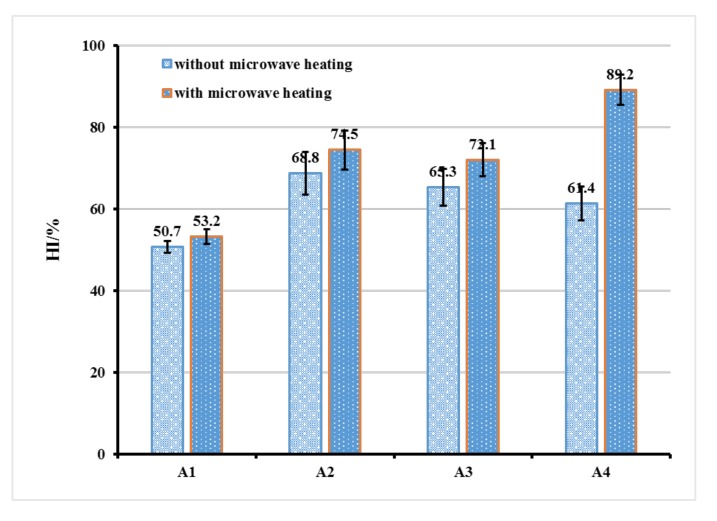
Self-healing capacity of asphalt mixture containing the fiber.

The self-healing capacity of asphalt mixture with the fiber was tested by 3PB experiment and the result is shown in Figure 9. As shown in Figure 9, the HI of all fiber containing asphalt mixtures increased to varying degrees. Under the rejuvenation effect of rejuvenator, the HI of A2, A3 and A4 increased from 50.7% to 68.8%, 65.3% and 61.4%, respectively. Under the synergistic action of microwave heating and rejuvenator, the HI of A2, A3 and A4 increased from 53.2% to 74.5%, 72.1% and 89.2%, respectively. The result revealed that the addition of three kinds of fiber could improve the self-healing ability of asphalt mixture under the action of rejuvenator that was encapsulated in fiber. Furthermore, because of the incorporation of microwave absorbent (GO) into self-healing fiber, under the synergistic action of microwave heating and rejuvenator, the self-healing ability of asphalt mixture containing MRF could be significantly improved. GO is an excellent microwave heating material that generates a large amount of heat under the action of microwave heating. As the temperature of the asphalt increases rapidly, the fluidity of the asphalt is greatly improved. Under the action of the rejuvenator and temperature, the crack inside the asphalt mixture will be quickly filled by the flowing asphalt, so the self-healing properties of the asphalt mixture containing Ca–alginate/GO fiber are significantly improved after microwave heating (as shown in Figure 9 A4).

## 4. Conclusions

In this study, three kinds of self-healing fiber were synthesized. A series of characterizations were performed to study the morphology, chemical structure and thermal stability of the fibers. Then, road performance and self-healing capacity of asphalt mixture containing the fiber were investigated. Based on the tests and results, the follow conclusions can be drawn:

(a) Ca–alginate fiber, Ca–alginate/SiO_2_ composite fiber and Ca–alginate/GO composite fiber were successfully synthesized by microfluidic device. Nano SiO_2_ and GO particles were incorporated into the fiber wall. Rejuvenator was encapsulated inside those fibers in the form of droplets. The three kinds of fibers had excellent thermal stability, which meet the temperature requirement in the mixing and compaction process of asphalt mixture.
(b) The addition of the three kinds of fibers improved high temperature anti-rutting ability of asphalt mixture. In addition, those fibers increased flexural strength and flexural modulus, while slightly decreased flexural strain of asphalt mixture. Moisture stability of asphalt mixture containing the fiber had a slight decrease. In addition, the fibers could prolong fatigue life of asphalt mixture under the action of encapsulated rejuvenator. In short, the road performances of asphalt mixture containing the fiber meet the requirements.
(c) The self-healing ability of asphalt mixture with fiber was better than that of asphalt mixture without the fiber. It was worth noting that the synergistic action of microwave heating and rejuvenator could further significantly improve the self-healing ability of asphalt mixture.

This study provides the possibility of industrial production of self-healing asphalt mixtures. Different types of self-healing fibers can be fabricated in large scale by this method. Personally, I believe that the future trend is a combination of capsule method and induction method. Combining the advantages of capsules method and induction heating method to achieve a substantial improvement in the self-healing properties of asphalt concrete. As in this work, the composite Ca–alginate/GO fibers with crack response behavior and microwave heating response behavior combined the advantages of the capsules method and induction heating method. The fiber not only can rejuvenate the asphalt, but also can rapidly increase the temperature of the asphalt by microwave heating, which quickly increased the fluidity of asphalt, and thus the self-healing properties of the asphalt mixture can be quickly improved under the synergistic effect of the rejuvenator and the temperature.

Highlights

- Three kinds of fibers encapsulating asphalt rejuvenator were synthesized;
- Road performance of asphalt mixture containing the fiber met the requirements;
- Three kinds of fibers could improve self-healing ability of asphalt mixture.

## Figures and Tables

**Table 1:** Aggregate gradation of AC-13 asphalt mixture.

Sieve Size/mm	Designed Gradation/%
16	100
13.2	96.2
9.5	75.2
4.75	47.4
2.36	30.8
1.18	23.9
0.6	16.6
0.3	12.3
0.15	9.1
0.075	6.9

**Table 2:** Types symbology of fibers and asphalt mixtures.

Types of Fiber	Symbology	Types of Asphalt Mixture	Symbology
Ca–alginate fiber	AF	Asphalt mixture containing Ca–alginate fiber	A2
Ca–alginate/SiO_2_ fiber	ASF	Asphalt mixture containing Ca–alginate/SiO_2_ fiber	A3
Ca–alginate/GO fiber	MRF	Asphalt mixture containing Ca–alginate/GO fiber	A4
